# Improvement of Quality Properties and Shelf Life Stability of New Formulated Muffins Based on Black Rice

**DOI:** 10.3390/molecules23113047

**Published:** 2018-11-21

**Authors:** Constantin Croitoru, Claudia Mureșan, Mihaela Turturică, Nicoleta Stănciuc, Doina Georgeta Andronoiu, Loredana Dumitrașcu, Vasilica Barbu, Elena Enachi (Ioniță), Georgiana Horincar (Parfene), Gabriela Râpeanu

**Affiliations:** 1 Academy of Agricultural and Forestry Sciences, 61 Marasti Blvd, 011464 Bucharest, Romania; c.croitoru@sodinal.com; 2 Faculty of Food Engineering, Tourism and Environmental Protection, Aurel Vlaicu University of Arad, 2 Elena Dragoi Street, 310330 Arad, Romania; claudia.muresan@uav.ro; 3 Integrated Center for Research, Expertise and Technological Transfer in Food Industry, Faculty of Food Science and Engineering, Dunarea de Jos University of Galati, 111 Domnească Street, 800201 Galati, Romania; mihaela.turturica@ugal.ro (M.T.); nsava@ugal.ro (N.S.); Georgeta.Andronoiu@ugal.ro (D.G.A.); ldumitrascu@ugal.ro (L.D.); vbarbu@ugal.ro (V.B.); elena.ionita@ugal.ro (E.E.); gparfene@ugal.ro (G.H.)

**Keywords:** black rice flour, anthocyanins, antioxidant activity, low gluten muffins, added value products

## Abstract

Effects of partial (50%) and total replacement of wheat flour with black rice flour on the phytochemical, physico-chemical, sensorial, and textural properties of muffins were studied. Partial or total replacement of wheat flour with black rice flour in muffins improved their nutritional and antioxidative properties with a positive effect on microbiological and color stability during the storage period in accelerated conditions. The low gluten muffins had an anthocyanin content of 27.54 ± 2.22 mg cyanidin-3-glucoside (C3G)/100 g dry weight (DW), whereas the gluten free muffins had 46.11 ± 3.91 mg C3G/100 g DW, with significant antioxidant values. Retention of 60% and 64% for anthocyanins and 72% and 80% for antioxidant activity after baking was found. The fracturability and hardness scores increased with the addition of black rice flour, whereas firmness and chewiness increased for gluten free muffins. The confocal analysis revealed a tendency of glucidic components to aggregate, with gathers of small bunches of black rice starch granules comprising anthocyanin. The results allowed designing two new value added bakery products, low and free gluten muffins, with significant high amounts of bioactive compounds, suggesting the functional potential of black rice flour.

## 1. Introduction

Muffins are sweet baked products highly appreciated by consumers due to their good taste and soft texture, perfect for breakfast, brunch and snacks. Muffin composition is a fat in water emulsion obtained from an egg-sugar-water-fat mixture as a continuous phase, and air bubbles represent a discontinuous phase where the flour is dispersed. Muffins are generally associated with a high porous spongy texture [1,2]. Traditionally, a muffin recipe is composed of wheat flour, vegetable oil, eggs and milk [3]. For this reason, many people with celiac disease are unable to consume this type of product since they are made with wheat flour.

The demand for low gluten and gluten-free products is increasing because it is well known that celiac disease is a common lifelong disorder, affecting 1% of the world’s population [4,5,6]. The reaction to gluten ingestion for those who sufferer from celiac disease is the inflammation of the small intestine leading to malabsorption of the nutrients [7,8]. However, a gluten free diet is characterized by low daily energy intake combined, with an unbalanced macronutrient content, compared to a balanced normal daily diet [9]. In avoiding the use of gluten in foods, significant technological and quality problems will have to be solved. Their sensorial properties are still different from similar products containing gluten. The main ingredients of gluten free cereal products are gluten-free flours, corn, rice and potato starches and different hydrocolloids that slow down the gluten viscoelastic properties. Recent studies were performed to improve the nutritional profile of gluten-free products by using pseudo-cereals as functional gluten-free ingredients [10,11,12]. In recent years, there have been many research projects for the development of gluten free sweet bakery products aimed to improve the organoleptic properties of the finished products [13,14].

Rice is a suitable cereal for developing gluten free products because it has a low level of prolamine and is hypoallergenic [15]. The main ingredient of the gluten free muffins, cake or cupcakes recipes is the rice flour [13,14,16], or different starch sources, such as corn, potato and wheat [17].

Rice flour has a big potential to be a wheat flour substitute in muffins because it has been used before to prepare gluten free bakery products, such as breads and cakes, which are traditionally made with wheat flour [8]. However, less information is available on the use of rice flour for gluten free products such as muffins. Several researchers have developed gluten free products using starches, dairy products, probiotics, gums and hydrocolloids to improve the structure and taste of the products [8,18].

Black rice was grown at a small scale in the early history of agriculture. In fact, black rice is considered to have the highest nutritional profile of all the cereals. The interest in black rice is growing because is gluten free, cholesterol-free, and low in sugar, salt and fat. Among these properties it also contains anthocyanins, antioxidants, B and E vitamins, iron, thiamine, magnesium, niacin and phosphorous and high fiber content. There are a lot of scientific studies showing that black rice powder is one of nature’s most well-balanced foods [19]. Black rice anthocyanins are about 26.3% and the most effective constituents in a percentage of 90% are represented by the cyanidin-3-*O*-glucoside and peonidin-3-*O*-glucosid anthocyanin [20]. Anthocyanins represent the flavonoid pigments of the black rice and they are a source of antioxidants that have the ability to inhibit the formation or to reduce the concentrations of reactive cell damaging free radicals [21].

The aim of the present study was to obtain value added low gluten and gluten-free black rice based muffins. In order to demonstrate the added-value of the products, the muffins were tested for total phyto-chemical, physico-chemical and microbiological properties, textural and sensorial analysis. An accelerated storage test for phytochemicals and microbiological stability was performed over 21 days at a temperature of 25 °C.

## 2. Results and Discussion

### 2.1. Black Rice Flour, Batter and Muffin Characterization

**Figure 1:**
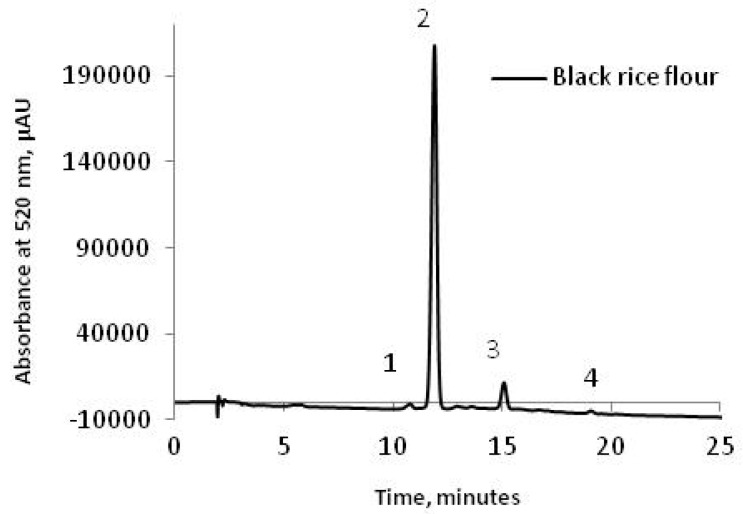
HPLC chromatogram of anthocyanins from black rice flour.

The black rice flours were characterized in terms of anthocyanins content using the chromatographic technique. Figure 1 shows a typical HPLC chromatogram, where the major anthocyanin found was cyanidin-3-glucoside. Four compounds were found in the black rice flour extract among which only three of them were identified as follows: Peak 1, cyanidin-3,5-diglucoside (1.08 mg/100 g dry weight (DW)); peak 2, cyanidin-3-glucoside (176.83 mg/100 g DW); peak 3, peonidin-3-glucoside (7.08 mg/100 g DW); and peak 4, unidentified. The total anthocyanin content in black rice flour was 192.36 ± 1.14 mg (C3G)/100 g DW. The results are similar with ones reported by Bordiga et al. [22] and Melini et al. [23] who studied the same variety of black rice. Bolea et al. [24] reported significant lower quantities of 21.00 µg/g cyanidin-3-glucoside and 0.10 µg/g peonidin-3-glucoside in the whole black rice flour.

Cyanidin-3-glucoside and peonidin-3-glucoside have been previously identified as the main anthocyanins present in the black rice [25,26]. According to other studies reported by Zhang et al. [27,28] five anthocyanins have been separated and identified in waxy and non-waxy black rice. These anthocyanins were malvidin, pelargonidin-3,5-diglucoside, cyanidin-3-glucoside, cyanidin-3,5- diglucoside and peonidin-3-glucoside.

The presence of gluten in black rice flour has been checked with a gluten ELISA assay and found no gluten presence in black rice flour.

The phytochemical characteristics of batters are described in Table 1. The physico-chemical and phytochemical characteristics of muffins are presented in Table 2. The total anthocyanin content (TAC) in S2 batter was 69.93 ± 2.34 mg cyanidin-3-glucoside (C3G)/100 g DW and 125.4 ± 6.64 mg C3G/100 g DW for S3 batter. After baking, the TAC was 27.54 ± 2.22 mg C3G//100 g DW for S2 muffins and 46.11 ± 3.91 mg C3G//100 g DW for S3 muffins, respectively. Therefore, the retention of TAC in the S2 and S3 after baking was approximately 60% and 64%. The total polyphenolic content (TPC) in batters were 254.1 ± 5.52 mg gallic acid (GA)/100 g DW and 307.3 ± 1.02 mg GA/100 g DW in S2 and S3, respectively, whereas baking caused a decrease to 170.3 ± 4.55 mg GA/100 g DW and 226.5 ± 2.14 mg GA/100 g DW.

Therefore, the retention of TPC in the S2 and S3 after baking was approximately 67% and 74%, respectively. Total flavonoid content (TFC) in batter were 149.4 ± 3.10 mg catechin equivalents (CE)/100 g DW and 187.1 ± 5.04 mg CE/100 g DW, respectively. Baking caused a slight decrease in TFC to 133.4 ± 1.88 mg CE/100 g DW and to 158.6 ± 1.02 mg CE/100 gDW, respectively, with a retention of 89% in S2 and 85% in S3. The antioxidant activities in batter were 611.2 ± 8.32 mM 6-Hydroxy-2,5,7,8-tetramethylchromane-2-carboxylic acid (Trolox)/100g DW and 687.72 ± 4.11 mM Trolox/100g DW for S2 and S3 respectively, whereas in muffins the corresponding values were 445.89 ± 2.22 mM Trolox/100g DW and 552.71 ± 5.06 mM Trolox/100g DW. Coefficients of 72% and 80% respectively were found for antioxidant retention in muffins after cooking.

However, it is difficult to estimate the effect of food matrices on the different phytochemicals, due to the complexity and different processing parameters. For example, retention of malvidin in the bun and biscuit after baking were 95.9% and 98.6%, respectively as reported by Karakaya et al. [29]. Similarly, a significant decrease ranging from 37.5% to 70% in the TAC content was determined during snack production by Nemś et al. [30], whereas Barti et al. [31] found a decrease in the anthocyanin content of breads produced using purple and blue wheat flours during the baking process. As expected regarding the colorimetric parameters, a lower *L* * value was observed for S3, whereas *a* * and *b* * values suggested a red dark (S2) to red brown (S3) color, with a pleasant taste due to the presence of black rice.

### 2.2. Sensory Analysis

Table 3 shows the average scores of sensorial attributes evaluated by the panelists.

Control muffins (S1) as expected showed the lightest color, while S3, was the darkest (*p* < 0.001). Surface humidity was perceived as being higher (*p* < 0.05) for S3 compared with S1 and S2. The scores given for fracturability and hardness attribute increased with the addition of black rice flour, reaching a maximum for S3.

The taste of all samples was appreciated; however the control sample was evaluated by the panelists with the highest score. Some panelists perceived that samples with black rice flour contained some crispy particles as compared with the control sample. Overall acceptability indicates that the panelists liked the analyzed muffins, regardless of whether or not they contained black rice flour. These results indicate that gluten free muffins obtained with black rice flour could be an alternative for people suffering from gluten intolerance. 

### 2.3. Texture Analysis

Texture parameters revealed by instrumental analysis are shown in Table 4. Firmness, defined as the maximum force required to compress the samples in the first cycle, varied between 4.75 ± 0.16 N for S1 and 6.67 ± 0.02 N for S3. Similar values for firmness were reported by Demirkesen et al. [32] and Wronkowska et al. [33] for bread formulated with rice, wheat, chestnut flour or buckwheat. The smaller value of control firmness could be explained by the presence of glutenin and prolamin (the major fractions of gluten) which are responsible for the porous network in muffins.

In the samples containing black rice flour, the reduced porosity led to a higher resistance during compression. Cohesiveness, determined as the ratio between the resistance of the samples during the second and the first compression, and springiness, defined as the deformation recovered between the two compression cycles, showed the highest values for S1 sample. These values may be due to the presence of glutenin, which is responsible for elastic and cohesive properties of dough [32]. Chewiness, described as the energy required to disintegrate the food during mastication, raised from 10.15 ± 0.23 mJ for S1 sample, to 12.21 ± 0.23 mJ for S2 sample and 15.13 ± 0.17 mJ for S3.

### 2.4. Confocal Microscopy Analysis

**Figure 2:**
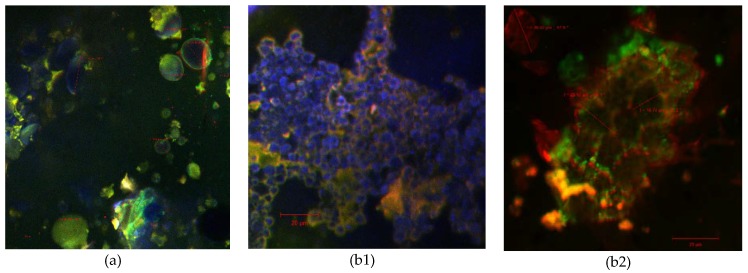
Confocal laser scanning microscopy images of control 1—wheat flour (**a**), and control 2—black rice flour (**b1** and **b2**).

The wheat flour that was analyzed as the first control sample (control 1) contained starch granules that can be grouped into three categories. The major category (about 60%) was displayed as large, lenticular or disc shaped granules with a diameter > 10 μm. Approximately 30% of the wheat starch granules were spherical, medium-sized (3–10 μm), while 10% were really small grains (under 3 μm) with irregular forms (as it can be seen in Figure 2a). The heterogeneity of the wheat flour starch granules could be attributed to the wheat variety (soft or hard wheat), the amylose content, and especially the moment in which is formed during anthesis or their different times of formation during grain development [34,35,36,37,38]. There are also many studies that confirm the presence of amylose in the peripheral region of the starch granules as it was likewise assessed in our study. As such, in Figure 2a it can be observed an interaction between the lipophilic dye molecules and some granules that afterwards displayed a green border whereas in the central location (in the hilum) longer amylopectin chains were noticed to form several inclusion complexes with the ligands as it was also suggested by Manca et al. [39].

The starch granule sizes of the black rice flower (control 2) were somewhere around 2–10 μm, similar to the results obtained by BeMiller & Whistler [40]. Rice starch granules were polygonal, irregular in shape [41] with sharp angles, and without any obvious concentric striations, hilum or cleft (Figure 2(b1)), most of them were grouped into large aggregates as can be seen in Figure 2(b2). The same characteristics were also reported by Leewatchararongjaroen & Anuntagool [42]. 

**Figure 3:**
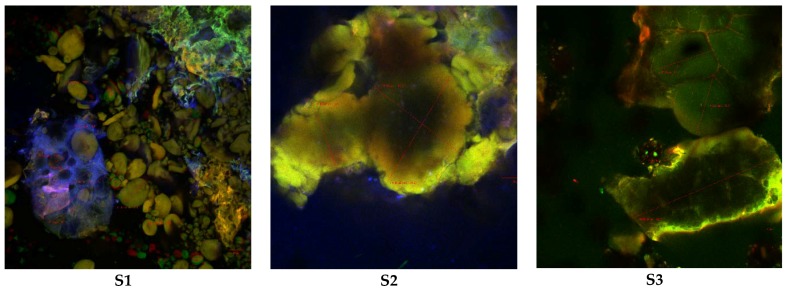
Confocal laser scanning microscopy images of muffin samples: (**S1**) (muffins with wheat flour), (**S2**) (muffins with 1:1 wheat and black rice flour) and (**S3**) (muffins with black rice flour).

The confocal analysis of the muffins samples displayed a much greater complexity due to the different biochemical composition of the ingredients that were used for the recipe (butter, sugar, eggs and wheat flour and black rice flour in variable proportions). It was more difficult to distinguish the components in the cooked samples, possibly as a result of the complex interactions between the gelatinized or expanded starch, denatured proteins and lipids. When only the wheat flour was used (S1 sample), in the texture of the baked dough, large wheat starch intact granules were also observed, the granules being isolated or grouped into the complex protein matrix. The size of the isolated particles was variable, from 9.56 to 43.44 μm, and the largest conglomerates exceeded 100 μm (Figure 3(S1)). By increasing the proportion of black rice flour, the glucidic components displayed a more obvious tendency towards aggregation so that around the large granules of the wheat starch gathered small bunches of black rice starch granules that come with the intake of anthocyanins (in green) (Figure 3(S2)). Confocal images taken for the muffins prepared with simple black rice flour frequently showed huge clusters (over 200 μm in size) consisting of starch granules, most of them having expanded due to the cooking temperature (about 80 μm in diameter) and at the same time being strongly colored in green due to the presence of anthocyanins, as it can be seen in Figure 3(S3).

Our results were similar to those obtained by Malik et al. [43]. It has been found that by replacing the wheat flour with rice flour in pastries or baked goods, the firmness and the sensorial attributes of freeze-thawed cake are improved due to a low amylose content of rice flour [44]. Furthermore, black rice flour also brings additional bioactive compounds such as anthocyanin pigments that are valuable in improving the food functionality.

### 2.5. Anthocyanin in Vitro Digestibility

**Figure 4:**
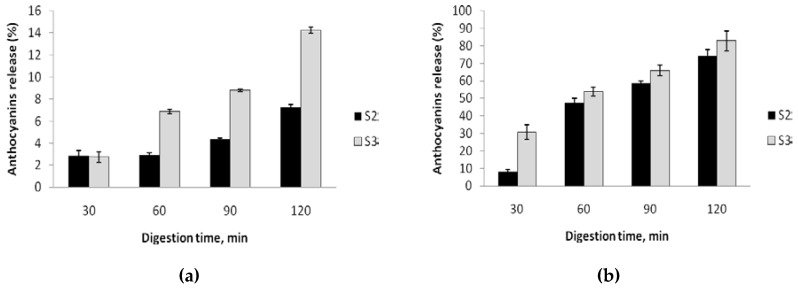
The patterns of gastric (**a**) and duodenal (**b**) digestion of formulated muffins S2 (muffins with 1:1 wheat and black rice flour) and S3 (muffins with black rice flour).

To evaluate the anthocyanins in vitro digestibity of new formulated muffins, simulated digestions conditions were applied. The digestion pattern of formulated muffins is given in Figure 4. As can be seen from Figure 4a, the maximum release of anthocyanins registered for the S3 sample was 14.23 ± 1.02% after 120 min of reaction. The digestion of S3 samples was limited with a maximum release of 7.22 ± 0.69% after 120 min of reaction. The results presented in Figure 4b during duodenal digestion revealed that the anthocyanin release was faster in the case of S3 compared with S2. From our results, it seems that less than 26% of the anthocyanins in S2 and 18% in S3 were retained in the formulated muffins during in vitro digestion.

Therefore, it can be appreciated that anthocyanins were slowly released from the muffins under simulated digestion conditions. Our results are similar with those reported by Sari et al. [45], suggesting that curcumin is released slowly from the nanoemulsion under simulated digestion conditions. Our in vitro digestibility results support a slowly release of anthocyanins from the food matrices during simulated gastric digestion and a significant release of the bioactive compounds into the gut.

### 2.6. Shelf-Life Assessment

**Figure 5:**
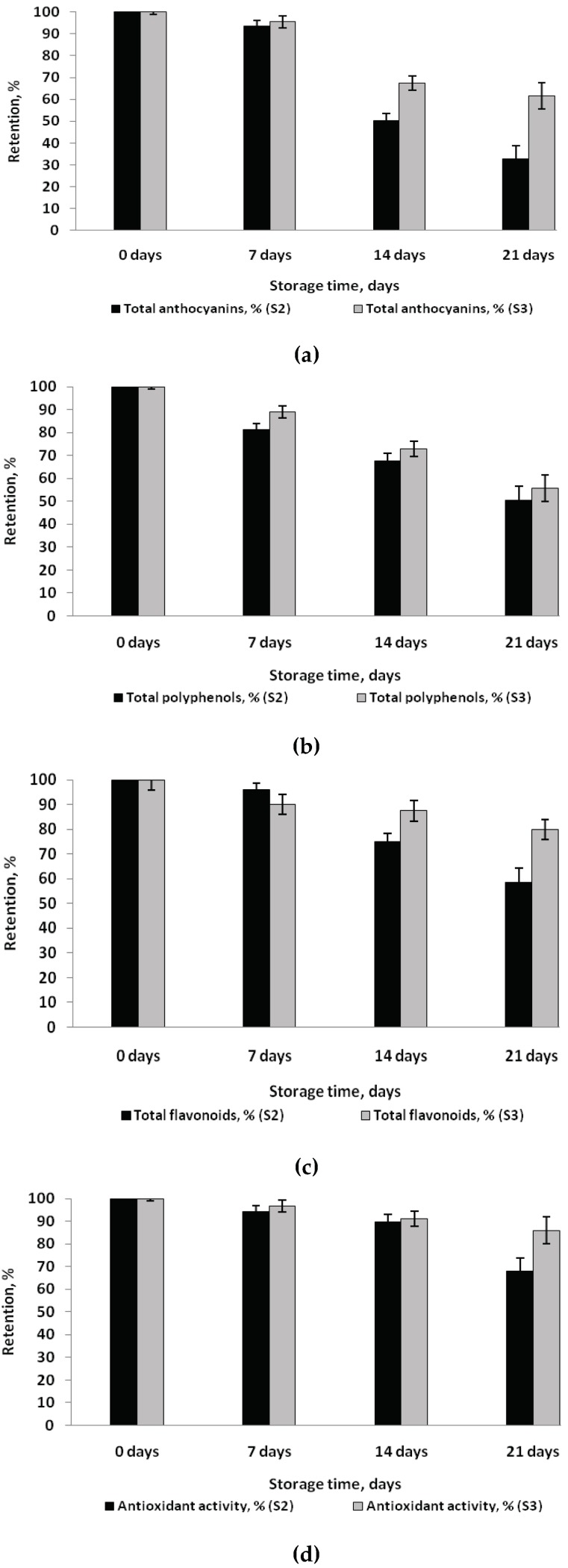
The retention in anthocyanis content (**a**), total polyphenols (**b**), total flavonoids (**c**) and antioxidant activity (**d**) of muffins during storage at a temperature of 25 °C for 21 days.

To evaluate the phytochemicals and antioxidant activity and color stability in the newly formulated matrix, the samples were stored at a temperature of 25 °C for 21 days. At every seven days, the following parameters were measured: Total polyphenolic, flavonoids and anthocyanins content, antioxidant activity, color parameters and molds and yeasts. Data from Figure 5 showed the total anthocyanin content, total polyphenols, total flavonoids content and antioxidant activity changes during the storage period. The anthocyanin content significantly decreased, up to 50% in S2 and 33% in S3 in the first 14 days of storage, whereas degradation continued up to 68% and 39%, respectively after 21 days, probably due to degradation reactions (Figure 5a).

It can be noticed that the anthocyanin’s degradation was more significant in S2 compared to S3, probably due to the higher concentration of the polyphenolic compounds, which exhibited a protective action. Regarding the total polyphenolic content in muffins during storage, the decreases were up to 50% (S2) and 45% (S3), and for flavonoids, up to 42% (S2) and 22% (S3) (Figure 5b,c). However, a slow decrease in antioxidant activity was found in all samples (Figure 5d). Therefore, after 14 days of storage, antioxidant activity decreases by 11% and 9% in S2 and S3, respectively, and by 33% and 15% after 21 days of storage. As expected, the decrease in antioxidant activity was lower in S3 when compared with S2, due to the higher concentration of polyphenolic compounds. However, it seems that the anthocyanin degradation does not significantly affect the antioxidant activity in all tested samples. Malvidin stability in the anthocyanin enriched bun after 21 days at room temperature was significantly lower than those of buns stored for 7 days. However, Karakaya et al. [29] reported that storage of 21 days at room temperature did not cause huge losses in anthocyanin contents of the bun and biscuits.

Table 5 shows the variation of color parameters. A slight increase in *L* * values can be observed with increasing storage time for all samples, likely due to anthocyanin degradation. Significant differences in brightness (*p* < 0.05) can also noticed, both in terms of samples and period of storage. The control sample (S1) had *a* * value close to 0 with no variation during storage time, while for S2 and S3 samples the *a* * value increased. Our results are in line with ones reported by Ursache et al. [46].

The *b* * values which denote a yellow color of the samples, had higher values for S1 and lower S3 baked with only black rice flour.

From microbiological point of view the results suggested that value-added muffins are microbiologically satisfactory during the accelerated storage test compared to control (Table 6).

## 3. Materials and Methods

### 3.1. Materials and Chemicals

2,2-Diphenyl-1-picrylhydrazyl (DPPH), 6-Hydroxy-2,5,7,8-tetramethylchromane-2-carboxylic acid (Trolox), Folin-Ciocalteu reagent, sodium carbonate, sodium hydroxide, sodium acetate, sodium nitrite, potassium chloride, aluminum chloride, gallic acid, catehine, potassium persulfate, formic acid, ethanol and methanol (HPLC grade), cyanidin and peonidin standards were obtained from Sigma Aldrich Steinheim, Germany. For muffin preparation, coconut butter with 80% fat content, brown sugar, hen eggs, wheat flour and black rice (*Oryza sativa L*. ssp. *Japonica*, Nerone variety, Italy) were purchased from the local supermarket, Galati, Romania.

### 3.2. Batters and Muffins Preparation

Preparation of the muffin batter was performed with the following steps: The coconut butter was mixed continuously with salt and brown sugar until the sugar is dissolved and a foam is formed; then the eggs were added, alternately with wheat flour (**S1 sample**, considered as control), wheat and black rice flour (1:1) (**S2 sample**), black rice flour (**S3 sample**), and finally the baking powder was added. The batter was mixed for 10 min at 300 rpm in order to get a uniform composition. Finally, the batter was filled in paper cups and baked at 185 °C for 25 min in a convection oven with forced air circulation. Products from each recipe were produced, baked and analyzed in two independent batches. The muffins were packed in vacuum bags at 800 mbar and stored at a temperature of 25 °C for 21 days.

### 3.3. HPLC Technique

The chromatographic analysis of the anthocyanins from black rice flour was performed as described earlier by Bolea et al. [24]. HPLC analysis was performed using a Surveyor HPLC system, controlled by an Xcalibur software system (Finnigan Surveyor LC, Thermo Scientific, Waltham, MA, USA). The anthocyanins detected in black rice flour were analyzed at a wavelength of 520 nm. The column used for this analysis was a C18 BDS Hypersil (150 mm × 4.6 mm, 5 mm). The gradient used for the elution of the anthocyanins was: 0–20 min: 9–35% (A), 20–30 min: 35% (A), 30–40 min: 35–50% (A), 40–45 min: 50–9% (A), with an injection volume of 10 µL, and the flow rate maintained at 1.000 mL/min.

### 3.4. Sensorial Analysis

A panel consisting of 11 different panelists aged between 29–50 years old performed the sensorial analysis of gluten free and added value muffins according to seven point hedonic scale. The panelists assessed the muffins samples for color (light to dark), surface humidity (none to very high), cross section appearance (non uniform to uniform), denseness (dense to airy), fracturability (low to very high), hardness (low to very hard), cohesiveness (none to tight mass), moistness of mass (low to very high), taste (dislike very much to like extremely), and overall likeability of the product (dislike to like extremely). Muffins samples were served in random order to panelists on white papers. Water was used for mouth rising before and between samples. 

### 3.5. Physico-Chemical, Phytochemical and Microbiological Analysis of Muffins

Standardized and validated laboratory methods were used to determine the physico-chemical characteristics of muffins, in terms of moisture, fat, protein, carbohydrates, ash and energy value.

The phytochemical content of extract (total polyphenols, total flavonoids, total monomeric anthocyanins) and antioxidant activity were determined as described by Turturică et al. [47]. In brief, 1 g of muffin was crushed and then mixed with 8 mL of 70% ethanol and 1 mL HCl 1N. The mixture was stirred for 8 h at room temperature on an orbital shaker at 150 rpm. After centrifugation at 6000 rpm for 10 min, the supernatant was collected and concentrated at 40 °C to dryness under reduced pressure (AVC 2-18, Christ, UK). The extracts were redissolved in 2 mL of MiliQ water and used for phytochemical analysis.

### 3.6. Microbiological Assessment

The microbiological shelf life of muffins was evaluated by monitoring fungal growth over 1, 7, 14 and 21 days of storage. The standard pour plate method described by Ursache et al. [46] was used to count the number of yeasts and molds. The results were expressed as CFU/g of sample. Each sample was analysed in duplicate on each day of storage.

### 3.7. Textural Analysis of Muffins

The textural analysis was achieved by the Texture Profile Analysis (TPA) Method, using the Brookfield CT3-1000 analyzer. The sample preparation consisted of removing the rind of the muffins and cutting the core in cube shapes with the sight length of 15 mm. Then, a double compression was applied at a distance of 10 mm, at a speed of 1 mm/s, with no holding time between the two compression cycles. The trigger load was 0.02 N and the load cell was 1000 g. The compression was performed using an acrylic cylinder (diameter ~24.5 mm, height ~35 mm) (TA11/1000). The data were recorded and processed using the TexturePro CT V1.5 software. For each sample, five tests were performed. The textural parameters determined by TPA were firmness, cohesiveness, springiness and chewiness.

### 3.8. Confocal Microscopy Analysis

The comparative confocal analysis of the samples was performed in order to capture the structural, textural and compositional changes of the experimental variants, while for the control samples simple wheat flour and black rice flour were used. The confocal microscope that was used for the analysis is a Zeiss Axio Observer Z1 inverted microscope model (LSM 710) equipped with a laser scanning system: Diode laser (405 nm), Ar laser (458 nm, 488 nm and 514 nm), DPSS (561 nm pumped solid state diodes), and HeNe-laser (633 nm). The strong anthocyanin absorption in the visible range was registered between 465 nm and 550 nm [48] with an in vivo peak, between 537 nm and 542 nm [49]. The distribution of the pigments into the protein matrix was observed at the excitation wavelength of 488 nm and by applying the FS38 filter, whereas the emission was collected between 500–600 nm. The powder that was stained with two dyes, DAPI (1 μg/mL) and Red Congo (40 μM), in a ratio of 3:1:1, was observed using a 40x apochromatic objective (numerical aperture 1.4) and the FS49 and FS15 filters. The 3D images were rendered and analyzed with ZEN 2012 SP1 software (Black Edition).

### 3.9. In Vitro Digestibility

In vitro digestibility was performed by using a method described by Oancea et al. [50]. Briefly, 1 g of muffins (S2 and S3) was mixed with Tris-HCl buffer (10 mM, pH 7.7). The gastric digestion was performed by the addition of a simulated gastric fluid (SGF), which consisted of porcine pepsin (40 mg/mL in 0.1 M HCl) that was added to the initial mixtures in a ratio of 0.5 g of pepsin per 100 g of sample and the pH was adjusted to 2.0 with 6 M HCl. Regarding the enteric digestion step, the simulated intestinal fluid (SIF) consisted of a mixture containing pancreatin (2 mg/mL) and afterwards the resulting mixture was neutralized to pH 5.3 with 0.9 M sodium bicarbonate. The pH of the system was adjusted to 7.0 with 0.1M NaOH, prior to the incubation of the samples for 2 h. The incubation was performed in an SI—300R orbital shaking incubator (Medline Scientific, Oxfordshire, UK), at 100 rpm and 37 °C. The total anthocyanin’s content of the samples was measured at every 30 min during the in vitro digestion.

### 3.10. Colorimetric Study

The color parameter values of the muffins were measured using the Minolta CR-410 Chroma Meter (Konica Minolta, Osaka, Japan) as described by Ursache et al. [46]. The results were expressed as *L* * (a lower value indicates a darker color, black: *L* * = 0 and white: L * = 100), *a* * (indicate the balance between red (>0), green (0) and blue (<0) color), and *b* * (the balance between yellow (>0) and blue (<0) color). All the measurements were performed in triplicates.

### 3.11. Storage Stability

No preservatives were used in the recipe formulation of the gluten free and added value muffins. Therefore, an accelerated storage stability test was performed during a period of 21 days at temperature of 25 °C. Duplicate samples were considered for determination of the molds and yeast, total polyphenolic content, total flavonoids and anthocyanins content, antioxidant activity and color at every 7 days.

### 3.12. Statistical Analysis of Data

Minitab 18 statistical processing software was employed to perform the statistical evaluation of the sensorial data. First, the data were checked for normality and homoscedasticity using the Ryan Joiner test and the Bartlett test. Then, one-way ANOVA was used to identify if panelists detect any differences between samples considering a significance level of 0.05. Post-hoc analysis via Dunnett multiple comparisons with a control were performed when appropriate. All data reported in this study represent the averages of duplicate analyses and is reported as mean ± standard error of the mean.

## 4. Conclusions

The muffins baked with black rice flour presented a high anthocyanin content and antioxidant activity compared with the control sample baked with wheat flour. The textural analysis suggested that the addition of black rice caused the increase of firmness, springiness and chewiness, while the cohesiveness was lower compared with the control sample and was related to a weaker binding between the constituents. Confocal images taken for the muffins baked with black rice flour showed huge clusters (over 200 μm in size) consisting of starch granules, most of them having expanded due to the cooking temperature (about 80 μm in diameter) and at the same time being strongly colored in green due to the presence of anthocyanins. Sensorial analysis showed that all samples were appreciated; some panelists even perceived that samples with black rice flour contain pleasant crispy particles compared with the control sample.

Storage stability of muffins revealed a decrease of anthocyanins, antioxidant activity and color parameters. The added value products showed a microbiological stability during the accelerated storage period, probably due to the presence of polyphenolic compounds. These results indicated that value added muffins obtained with black rice flour could be an alternative for people suffering from gluten intolerance, whereas proving a significant amount of polyphenolic content, with potentially beneficial effects on human health.

## Figures and Tables

**Table 1:** Phytochemical characteristics of batters.

Phytochemical Properties	Samples		
	S1	S2	S3
Total anthocyanin content (TAC), mg cyanidin-3-glucoside (C3G)/100 g dry weight (DW)	n.d.	69.93 ± 2.34 ^a^	125.4 ± 6.64 ^b^
Total polyphenolic content (TPC), mg gallic acid (GA)/100 g DW	82.1 ± 1.06 ^a^	254.1 ± 5.52 ^b,c^	307.3 ± 1.02 ^b^
Total flavonoid content (TFC), mg catechin equivalents (CE)/100 g DW	71.2 ± 1.44 ^a^	149.4 ± 3.10 ^b^	187.1 ± 5.04 ^c^
Antioxidant activity, mM 6-Hydroxy-2,5,7,8-tetramethylchromane-2-carboxylic acid (Trolox)/100 g DW	152.8 ± 2.10 ^a^	611.2 ± 8.32 ^b,c^	552.71 ± 5.06 ^c^

* Values with different letters in the same raw are significantly different (*p* < 0.05).

**Table 2:** Physico-chemical and phytochemical characteristics of muffins.

Physico-Chemical and Phytochemical Properties		Samples		
		S1	S2	S3
Proteins, g/100 g		11.69 ± 0.57 ^b^	12.16 ± 1.16 ^a^	12.71 ± 0.92 ^a^
Fats, g/100 g		20.17 ± 1.37 ^b^	20.22 ± 0.45 ^c^	18.37 ± 1.91 ^a^
Carbohydrates, g/100 g		45.44 ± 2.60 ^a^	42.91 ± 1.68 ^b,c^	42.38 ± 2.51 ^c^
Moisture, g/100 g		20.60 ± 0.11 ^b^	22.53 ± 0.23 ^c^	24.13 ± 0.15 ^d^
Ash, g/100 g		2.10 ± 0.01 ^a^	2.18 ± 0.01 ^a^	2.41 ± 0.01 ^b^
Energy value, %: kcal kJ		421.81 1763.18	413.83 1729.82	396.71 ^a^ 1658.24 ^a^
TAC, mg C3G/100 g DW		n.d.	27.54 ± 2.22 ^a^	46.11 ± 3.91 ^b^
TPC, mg GA/100 g DW		64.4 ± 3.16 ^a^	170.3 ± 4.55 ^b^	226.5 ± 2.14 ^v^
TFC, mg CE/100 g DW		57.2 ± 0.94 ^a^	133.4 ± 1.88 ^b^	158.6 ± 1.02 ^c^
Antioxidant activity, mM Trolox/100 g DW		124.6 ± 3.20 ^a^	445.89 ± 2.22 ^b,c^	552.71 ± 5.06 ^c^
Colorimetric parameters	*L* *	80.41 ± 9.13 ^a^	27.71 ± 0.15 ^b^	19.6 ± 3.58 ^b,c^
	*a* *	0.06 ± 0.001 ^a^	8.47 ± 1.08 ^b^	6.53 ± 0.95 ^c^
	*b* *	51.83 ± 1.15 ^a^	7.31 ± 0.41 ^b^	1.49 ± 0.14 ^c^

* Values with different letters in the same raw are significantly different (*p* < 0.05).

**Table 3:** Sensory characteristics of muffins.

Sensorial Attribute	Samples		
	S1	S2	S3
Color	1.82 ± 0.87 ^a^	5.63 ± 1.2	6.27 ± 1.27
Surface humidity	3.27 ± 1.84 ^a^	3.82 ± 0.98 ^a^	4.72 ± 1.19
Cross section appereance	1.73 ± 1.10	1.64 ± 0.92	2.55 ± 1.7
Denseness	2.82 ± 1.47	2.82 ± 1.25	2.82 ± 1.94
Fracturability	2.46 ± 1.7	2.82 ± 1.33	3.64 ± 1.7
Hardness	2.64 ± 1.57 ^a^	3.73 ± 1.35 ^a^	4.36 ± 1.5
Cohesivity	5.46 ± 1.21	4.73 ± 1.00	4.55 ± 1.44
Moistness of mass	3.36 ± 1.75	3.46 ± 1.58	3.81 ± 2.27
Taste	6.00 ± 0.89	5.09 ± 1.22	4.90 ± 1.38
Sweetness	4.90 ± 1.51	4.27 ± 1.67	4.63 ± 1.7
Overall acceptability	5.90 ± 0.83	5.18 ± 0.98	5.18 ± 1.4

^a^ Based on Dunnett multiple comparisons with a control, means on the same row that do not share a letter are significantly different (*p* < 0.05).

**Table 4:** Texture parameters of muffins.

Textural Parameters, Unit	Samples		
	S1	S2	S3
Firmness, N	4.75 ± 0.16 ^a^	5.82 ± 0.26	6.67 ± 0.02
Cohesiveness, dimensionless	0.37 ± 0.01	0.35 ± 0.02	0.33 ± 0.02
Springiness, mm	6.95 ± 0.06	6.83 ± 0.08	6.57 ± 0.23
Chewiness, mJ	10.15 ± 0.23	12.21 ± 0.25	15.13 ± 0.17

^a^ Mean of the five determinations ± standard deviation.

**Table 5:** Colorimetric analysis of muffins.

Storage Period, Days	S1			S2			S3		
	Colorimetric Parameters								
	L *	a *	b *	L *	a *	b *	L *	a *	b *
**0**	80.41 ± 9.13 ^c,d^	0.06 ± 0.001 ^b,c^	51.83 ± 1.15 ^a,b^	27.71 ± 0.15 ^b,c,d^	8.47 ± 1.08 ^a,b,c,d^	7.31 ± 0.41 ^a,b,c^	19.6 ± 3.58 ^c^	6.53 ± 0.95 ^a b^	1.49 ± 0.14 ^a,b,c^
**7**	88.02 ± 0.83 ^a^	0.10 ± 0.001 ^b^	60.11 ± 4.96 ^b,c^	29.42 ± 0.30 ^d^	9.10 ± 1.63 ^a,b^	8.40 ± 0.83 ^a^	23.9 ± 1.96 ^b,c^	7.30 ± 1.30 ^a,b,d^	1.71 ± 0.26 ^a,b,c^
**14**	110.41 ± 5.25 ^a,b,c,d^	0.18 ± 0.011 ^b^	73.25 ± 2.49 ^d^	35.80 ± 73.74 ^a,b^	10.30 ± 1.84 ^a^	9.20 ± 0.94 ^c^	28.8 ± 2.49 ^d^	11.6 ± 0.22 ^c^	2.25 ± 0.66 ^a^
**21**	119.63 ±4.60 ^b,c,d^	0.21 ± 0.057 ^b^	75.53 ± 4.31 ^a,b^	38.51 ± 1.41 ^a,b,c^	11.11 ± 1.10 ^a,b,c,d^	9.62 ± 0.31 ^b,c^	30.1 ± 3.63 ^a,b^	13.2 ± 0.95 ^a,b^	3.51 ± 0.51 ^b,c,d^

Values with different letters in the same column are significantly different (*p* < 0.05) (L *—lightness, a *—redness, b *—yellowness).

**Table 6:** Yeasts and molds during storage (colony forming unit CFU/g).

Samples	Storage Period, Days			
	0	7	14	21
**S1**	<10	1.33∙× 10^2^ ± 0.13	2.59 × 10^3^ ± 0.08	5.16 × 10^5^ ± 1.10
**S2**	<10	<10	<100	<100
**S3**	<10	<10	<10	<100
